# A Fault Prediction and Cause Identification Approach in Complex Industrial Processes Based on Deep Learning

**DOI:** 10.1155/2021/6612342

**Published:** 2021-03-05

**Authors:** Yao Li

**Affiliations:** School of Computer Science and Engineering, Northeastern University, Liao Ning, China

## Abstract

Faults occurring in the production line can cause many losses. Predicting the fault events before they occur or identifying the causes can effectively reduce such losses. A modern production line can provide enough data to solve the problem. However, in the face of complex industrial processes, this problem will become very difficult depending on traditional methods. In this paper, we propose a new approach based on a deep learning (DL) algorithm to solve the problem. First, we regard these process data as a spatial sequence according to the production process, which is different from traditional time series data. Second, we improve the long short-term memory (LSTM) neural network in an encoder-decoder model to adapt to the branch structure, corresponding to the spatial sequence. Meanwhile, an attention mechanism (AM) algorithm is used in fault detection and cause identification. Third, instead of traditional biclassification, the output is defined as a sequence of fault types. The approach proposed in this article has two advantages. On the one hand, treating data as a spatial sequence rather than a time sequence can overcome multidimensional problems and improve prediction accuracy. On the other hand, in the trained neural network, the weight vectors generated by the AM algorithm can represent the correlation between faults and the input data. This correlation can help engineers identify the cause of faults. The proposed approach is compared with some well-developed fault diagnosing methods in the Tennessee Eastman process. Experimental results show that the approach has higher prediction accuracy, and the weight vector can accurately label the factors that cause faults.

## 1. Introduction

In the modern manufacturing industry, most production processes can be viewed as a continuous rolling process, such as assembly/product lines. Sometimes, unexpected faults occur in control or manufacturing systems, and the entire process will break down. Before the faults are found and fixed, many costs are wasted. The cost of wasted energy, resources, and time is significant, especially for high energy consumption process industries. Therefore, fault diagnosis and prognosis have been a subject of intensive research in the past four decades [1]. There are generally two research directions to solve this problem: first, detecting or predicting faults before they break the process, which will help workers or engineers prepare for production breaks in advance and yield great cost savings, and, second, identifying the causes and improving the production process, which can reduce the occurrence of breaks. With the development of the Industrial Internet of Things (IIoT), we can collect almost all of the production process data, which can be used to predict faults and identify causes. While these two directions may be easy to implement in simple industrial processes, there are still serious challenges in complex industrial processes, especially in complex process industries.

Challenges proposed by complex industries in related research are reflected in the substantial volume and high-dimensional input data. These data, referred to as big data, are generated from sensors, production equipment, and testing instruments. In complex process industries, it is common to generate data with thousands of dimensions, even without considering video stream data. These data include control parameters of production equipment, real-time production data, environmental perception, and inspection data. For example, for a medium-sized pulp-and-paper mill, a typical process industry, its entire production process includes 19 processes, 4 key raw materials, and two waste removals. The equipment, instruments, and sensors involved in the production process can generate more than 2000 kinds of data, and the volume will continue to grow over time. Facing high-dimensional and continuous growing data, machine learning (ML) algorithms can continuously improve performance. Therefore, ML, mainly deep learning (DL) and neural networks, are widely used in big data processing [2], including fault detection based on industrial big data.

Traditional DL-based algorithms consider the input data as time series data, which means that an input item x^(*i*)^ is the data generated by the entire production line at time *t*^(*i*)^, and the next input item x^(*i*+1)^ is the data at time *t*^(*i*+1)^. Afterwards, a DL algorithm, similar to a recurrent neural network (RNN), can be used, such as gate recurrent units (GRUs) and long short-term memory (LSTM). This is very intuitive because the data collected from the production process are arranged in chronological order. However, because the sampling frequency of the data in each dimension is different, the data obtained at different times from the production line is not comprehensive, which brings difficulties to the construction of a DL model.

In the actual production process, the faults that caused production breaks generally occurred at a previous time, and it is difficult for engineers to identify this time. For example, in the fused magnesia industry, a typical high-energy-consuming complex process industry, the underburning condition of the furnace is a common fault, which will cause the furnace to fail and break production, but the duration before the break is difficult to identify. However, a DL-based model needs this time to label the training data. Traditional DL-based fault detection approaches may have a good performance in some applications [3], but they cannot help engineers find the cause of the faults.

In this paper, we regard these process data as a sequence in space according to the production process and propose an improved LSTM neural network. Afterwards, an encoder-decoder framework and an attention mechanism (AM) algorithm are used to predict faults before they occur. The input is a sequence in which different types of data are arranged, according to the position of the production process.

The output is still a sequence arranged by different fault types and is specific to a certain output item. Its value represents the length of time before fault occurrence. This approach has three advantages: (1) the method can handle long spatial sequences and improve prediction accuracy. (2) Weight vectors in AM can indicate the correlation between faults and input data. It should be noted that when the input data is expressed in a time series, this correlation cannot be reflected. (3) The format of the output sequence can facilitate the labelling of the training data. At last, the proposed approach is evaluated on the Tennessee Eastman Process (TEP) [4, 5]. The main contributions of this paper can be summarized as follows:

1. The weight vectors of AM in the trained neural network are firstly used in fault diagnosis to reflect the correlation between faults and input data. This can help engineers find the cause of faults and improve the production process.
2. Different from the traditional DL model, industrial production data are treated as a time series, and we regard industrial production data as a spatial sequence according to the production process and propose a branched LSTM structure.
3. We designed the output as a fault type sequence. The value of a specific item represents the length of time before fault occurrence. This output model provides convenience for labelling training data.

Experiments show that our approach can achieve a higher accuracy in fault detection than other traditional methods. Moreover, the specific factors causing the faults can be identified.

The rest of this paper is organized as follows: Section 2 gives brief reviews of related works. In Section 3, we describe the problem statement and provide some assumptions. Afterwards, Section 4 gives the algorithm details: an improved LSTM-based encoder-decoder model is introduced and describes an AM algorithm for identifying factors. In Section 5, we test the fault detection approach and evaluate its performance. Finally, Section 6 gives the conclusion and direction of future work.

## 2. Related Works

Fault prediction or diagnosis is the process of detecting (or predicting) deviations from normal or expected operation [6]. Fault diagnosis has been widely used in industries for cost saving and safe production, and its applications are growing with the development IIoT and CPS. Therefore, it has long been attractive to many researchers.

Statistical analysis techniques are popular traditional signal processing methods, and there are three algorithms commonly used for fault detection: principal component analysis (PCA) [7], independent component analysis (ICA) [8], and partial least squares (PLS) [9, 10]. The core idea of PCA is to take the direction of multidimensional data with the largest variance as the main feature and make them have no correlation in different orthogonal directions. This is suitable for fault detection based on multivariate time series (MTS) data. For example, the authors in [11] coupled PCA with a Kalman filter to improve fault detection accuracy, and the key operation was to project the subspace along the fault area. The ICA algorithm considers the data to be linear combinations of statistically independent components. It is a demixing process. PLS is a supervised method that includes the ideas of PCA and canonical correlation analysis. This type of technique has its own limitations in processing these nonlinear MTS and imbalance data [12].

Deep learning is a powerful tool, and it has been successfully applied in many fields [13–15]. A report mentions that advances in DL techniques are the main enablers of knowledge work automation [16]. MTS data is a sequence model, so the commonly used DL is a recurrent neural network (RNN), mainly the LSTM model [12]. For example, Park et al. developed an LSTM-based fault detection model, called LiReD [17]. They did not focus on how to process the multidimensional input data but on edge computing. Lu et al. introduced an LSTM network to solve the early fault detection problem in high-dimensional sequential data [18]. LSTM has an efficient performance for sequential data processing, and it has been applied to fault detection models in many industries [19–24].

In the industrial production process, it should be noted that fault cases are rare, and, accordingly, the obtained training data contains a few fault examples. This is a class-imbalanced problem, and the proposed approach will also face this problem. There are three basic methods in class-imbalance learning: (1) undersampling [25], (2) synthetic minorities [26], and (3) cost-sensitive learning [27]. There are already well-developed solutions, so we will not go into details in this article.

Identifying in a fault detection algorithm the factors recorded by sensors that cause faults is valuable for industries. However, such studies are still scarce. An attention mechanism (AM) was originally used to ease the complexity of neural network models [28], and it is not necessary to input all information to the neural network for calculation, but only to select some task-related information for input into the neural network [29]. AM was primarily used for natural language recognition [30], but it was soon applied in the field of image-based deep learning [31, 32]. For example, it has proven to be a very effective tool in a variety of applications such as reading comprehension, abstractive summarization, textual entailment, and learning task-independent sentence representations [33–35].

In this paper, we proposed a branched LSTM structure to adapt to the spatial data structure generated by industrial production lines. Moreover, AM was firstly used in the encoder-decoder model for fault detection to improve accuracy. The most important is that weight vectors of AM will be used to represent the attention distribution, which can help engineers to identify the specific factors that cause the faults.

## 3. Problem Statement and Assumptions

### 3.1. Problem Structure

Data comes from a multivariate time series process and is collected by a large number of various types of sensors, equipment, and instruments in manufacturing. They are the inputs for the encoder-decoder model. Training data contains regular time-intervals (*X*) and the event label (*y*). The primary purpose of fault prediction is to build a classification model for different fault types and identify factors causing the faults.

**Figure 1:**
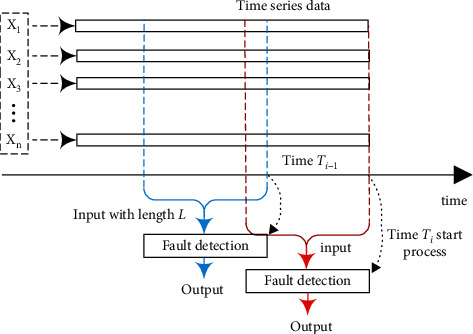
Fault detection process structure in time series data.

The sensing data increases with time. For this sequence of data, we start the fault detection program with a certain frequency and then use the current time and data from a previous period as input. In other words, the output of the model at time *T*_*i*−1_ will not be used as the input at time *T*_*i*_. The process is shown in Figure 1, where *T*_*i*_  −  *T*_*i*−1_ is the time interval for program startup and *L* is all sampled data at time length *L*.

The input data is a sequence. Each individual item in the sequence represents independent data collected from a certain position in the production process. We will describe in detail the structure of these items and how they are integrated in the encoder-decoder framework in the next section. The value of a single item is a data vector (time series) collected from a sensor, equipment, or instrument in the production line over a period of time *L*.

Because the underlying structure is different, the sampling frequency and data type of the items in the sequence are different, which means that the data in each item will be different in length, type, and so on. Accordingly, one of the training datasets can be described as {*X*^(1)^, *X*^(2)^,…, *X*^(*i*)^,…, *X*^(*n*)^, *Y*^(1)^, *Y*^(2)^,…, *Y*^(*i*)^,…, *Y*^(*m*)^}, where *X*^{(*i*)}^ is the input data from position *i* in the production line, and {*Y*^(1)^, *Y*^(2)^,…, *Y*^(*m*)^} is a class label matrix (output), which indicates the length of time before fault occurrence. The length of each *X*^(*i*)^ depends on *L*, and it refers to the sampling frequency in position *i*. The length of each *Y*^(*j*)^ depends on the type of faults.

According to the above description, the problem of fault prediction in the industrial production process can be regarded as a sequence-to-sequence (seq2seq) classification problem. The encoder-decoder model can then be used.

### 3.2. Assumptions

Industrial data used for fault detection is recorded by sensors, equipment, and instruments. The cause of these data anomalies may be faults in the production process or sensor failure. We focus on detecting or predicting faults in production in this paper, so we do not consider sensor failure. In addition, in the process of building a neural network, some basic operations are also involved to improve model performance, such as regularization and normalization. These operations are well-developed and popular technologies. Therefore, we will not describe them in detail in this paper.

The raw data collected from the production line is very rough. Generally, some simple algorithms can be used to reduce dimensionality. For example, a timestamp may be described as a six-dimensional vector, including year, month, day, hour, minute, and second. It can be easily integrated into a one-dimensional scalar. This situation is common in raw data, and it can be easily integrated according to the logical relationship. This integration algorithm is very simple and needs to be completed according to the actual situation. This article assumes that all input data has undergone such processing. However, readers need to pay attention to this step when using this algorithm and cannot be ignored.

## 4. Architecture and Algorithms

The architecture of the proposed approach works in an LSTM-based encoder-decoder model, and AM is used to improve fault detection accuracy and identify specific factors causing faults.

### 4.1. Input Sequence and Improved LSTM Structure

**Figure 2:**
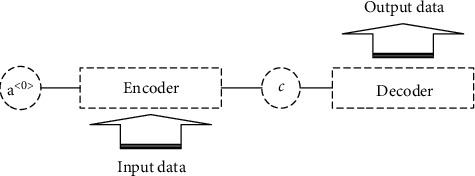
Typical encoder-decoder model.

A typical encoder-decoder model solves a seq2seq problem. It is a multi-input multioutput model, also known as many-to-many. The structure is illustrated in Figure 2.

According to the description above, the input sequence can be described as *X*^(1)^, *X*^(2)^,…, *X*^(*i*)^,…, *X*^(*n*)^, where *X*^(*i*)^ means a time series data from the position *i* in the production line. *X*^(*i*)^ can be described as

(1) $$\begin{array}{c} X^{\left(i\right)}=\left(x_{1}^{i},x_{2}^{i},\ldots ,x_{j}^{i},\ldots ,x_{m_{i}}^{i}\right), \end{array}$$

where *x*_*j*_^*i*^ means data from the position *i* at time *j*. And *m*_*i*_ is the length of *x*_*j*_^*i*^, meaning the number of data generated at position *i* over a period of time *L*, and it is related to the sampling frequency.

**Figure 3:**
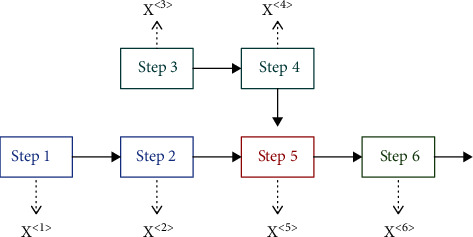
A simple production example.

In the actual production process, the production line is not a simple one-dimensional sequence. There are usually branches, which make it more complicated than the traditional seq2seq problem.  Figure 3 introduces a simple production example. The entire production process contains 6 steps, and each step generates production data *X*^(*i*)^. As shown in the figure, they are not a simple one-dimensional sequence. There is a branch at Step 5, which can execute itself only after Steps 2 and 4 are executed in parallel. As a result, the spatial structure of collected data {*X*^(1)^, *X*^(2)^,…, *X*^(*i*)^,…, *X*^(*n*)^} is not a simple one-dimensional sequence. Thus, we improved the LSTM-based encoder structure based on the spatial structure.

**Figure 4:**
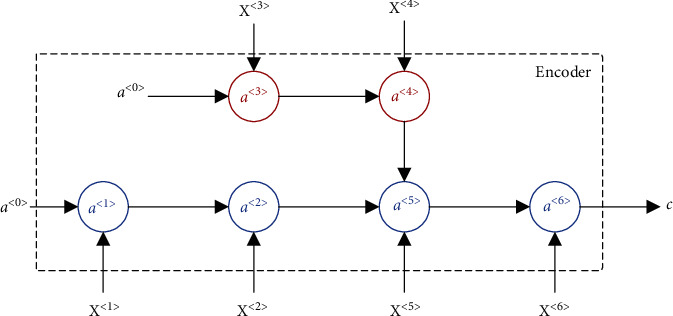
Branched LSTM chain in the encoder.

According to the spatial structure, we design a branched LSTM chain, which is illustrated in Figure 4. Each arrow in Figure 4 means a mapping between the different layers of the neural network. Accordingly, *a*^〈*i*〉^ and *C* are the outputs from the previous layer of the neural network. In this encoder structure, there are two situations: one is a traditional LSTM cell and the other is a cell with branches, which will be described separately below.

At first, for a traditional LSTM cell, *a*^〈*i*〉^ can be described as

(2) $$\begin{array}{c} a^{\langle i\rangle }=g_{i}\left(w_{\left(aa\right)}*a^{\langle i-1\rangle }+w_{\left(xa\right)}*X^{\left(i\right)}+b\right), \end{array}$$

where *g*_*i*_ is the activation function, *w*_(*aa*)_ is the weights matrix for the output of the previous layer, *w*_(*xa*)_ is the weights matrix for the input, and *b* is the bias.

**Figure 5:**
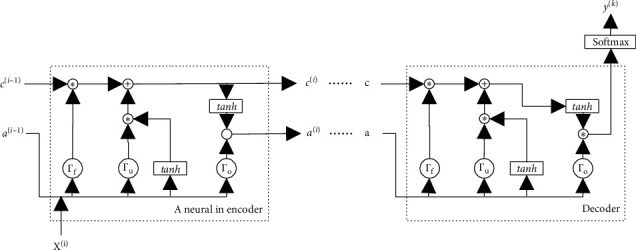
Traditional LSTM unit structure.

In industrial production, there is a deep connection in the time series for fault detection, and the LSTM model is capable of capturing this connection. The LSTM model was proposed by Sepp Hochreiter and Jiirgen Schrnidhuber in 1997 [36]. Compared to a Recurrent Neural Network (RNN), an LSTM cell contains three special-purpose gates for storing and selecting information, and there is a memory value between cells. The details are shown in Figure 5.

Γ_*f*_ is the forget gate. According to the input *a*^(*i* − 1)^ and *X*^(*i*)^, the forget gate can determine which information can be “forgotten.” It can be expressed as

(3) $$\begin{array}{c} \Gamma_{f}=\sigma \left(W_{f}^{\langle i\rangle }\;\left[a^{\langle i-1\rangle },X^{\langle i\rangle }\right]+b_{f}^{\left(i\right)}\right), \end{array}$$

where *a*^〈*i* − 1〉^ is the output of the previous LSTM cell, *X*^〈*i*〉^ is the data at time *i*, and *W*_*f*_^(*i*)^ is the weights matrix. After the *sigmoid* function, information with dimensions close to 0 will be “forgotten.”

The update gate is Γ_*u*_, and it can determine which information can be “added.” Γ_*o*_ is the output gate. They can be expressed as

(4) $$\begin{array}{c} \Gamma_{u}=\sigma \left(W_{u}^{\langle i\rangle }\;\left[a^{\langle i-1\rangle },X^{\langle i\rangle }\right]+b_{u}^{\left(i\right)}\right),\\ \Gamma_{o}=\sigma \left(W_{o}^{\langle i\rangle }\;\left[a^{\langle i-1\rangle },X^{\langle i\rangle }\right]+b_{o}^{\left(i\right)}\right). \end{array}$$

The memory cell *c*^(*i*)^ and the activated vector *a*^〈*i*〉^ can be expressed as

(5) $$\begin{array}{c} \bar{c^{\left(i\right)}}=\text{tan}h\left(W_{c}^{\left(i\right)}\;\left[a^{\langle i-1\rangle },X^{\langle i\rangle }\right]+b_{c}^{\left(i\right)}\right),\\ c^{\left(i\right)}=\Gamma_{u}*\bar{c^{\left(i\right)}}+\Gamma_{f}*c^{\left(i-1\right)},\\ a^{\left(i\right)}=\Gamma_{o}*c^{\left(i\right)}, \end{array}$$

where tan*h* is a hyperbolic tangent function:

(6) $$\begin{array}{c} \text{tan}h\left(x\right)=\frac{e^{x}-e^{-x}}{e^{x}+e^{-x}}. \end{array}$$

*X*^*i*^ in the equation above is an *m*_*i*_-dimensional vector, and *m*_*i*_ is the number of sensors in production. Accordingly, *W*_*u*_^(*i*)^, *W*_*f*_^(*i*)^, and *W*_*o*_^(*i*)^ are an *n* × (*n*+*m*_*i*_)-dimensional matrix, where *n* is the number of cell in the hidden layer. *b*_*u*_, *b*_*f*_, and *b*_*o*_ are *n*-dimensional vectors, and so is *c*^(*i*)^ and *a*^(*i*)^. The dimension of the weights matrix within each cell is related to the length of the input vector, so the cell can uniformly output a vector *a* with length *n*.

**Figure 6:**
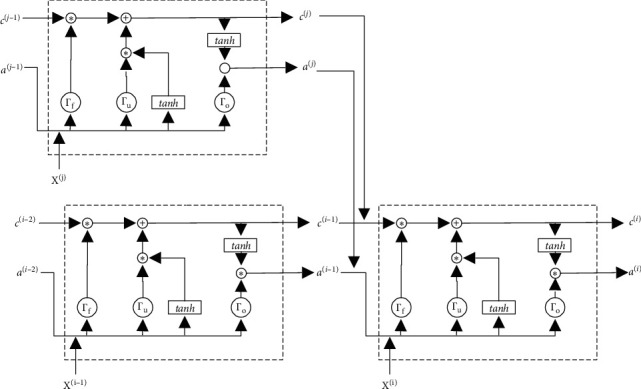
LSTM unit structure used in this paper.

Secondly, for a branched LSTM cell, an LSTM unit structure is illustrated in Figure 6. We suppose the cell of the other branch is *j*. Accordingly, based on the traditional LSTM cell [36], the key calculation process is modified as follows:

(7) $$\begin{array}{c} a^{\langle i\rangle }=g_{i}\left(w_{\left(aa\right)}*a^{\langle i-1\rangle }+w_{\left(aa_{j}\right)}*a^{\langle j\rangle }+w_{\left(xa\right)}*X^{\langle i\rangle }+b\right), \end{array}$$

where *a*^(*j*)^ is the output of a branch LSTM cell. Thus, the forget gate, update gate, and output gate can be expressed as

(8) $$\begin{array}{c} \Gamma_{f}=\sigma \left(W_{f}^{\left(i\right)}\;\left[a^{\langle i-1\rangle },a^{\langle j\rangle },X^{\langle i\rangle }\right]+b_{f}^{\left(i\right)}\right),\\ \Gamma_{u}=\sigma \left(W_{u}^{\langle i\rangle }\;\left[a^{\langle i-1\rangle },a^{\langle j\rangle },X^{\langle i\rangle }\right]+b_{u}^{\left(i\right)}\right),\\ \Gamma_{o}=\sigma \left(W_{o}^{\langle i\rangle }\;\left[a^{\langle i-1\rangle },a^{\langle j\rangle },X^{\langle i\rangle }\right]+b_{o}^{\left(i\right)}\right). \end{array}$$

The memory cell *c*^(*i*)^ and the activated vector *a*^〈*i*〉^ can be expressed as

(9) $$\begin{array}{c} \bar{c^{\left(i\right)}}=\text{tan}h\left(W_{c}^{\left(i\right)}\;\left[a^{\langle i-1\rangle },X^{\langle i\rangle }\right]+b_{c}^{\left(i\right)}\right),\\ c^{\left(i\right)}=\Gamma_{u}*\bar{c^{\left(i\right)}}+\Gamma_{f}*\left(\sigma \left(W_{c}^{\left(i\right)}\right)\;\left[c^{\left(i-1\right)},c^{\langle j\rangle }\right]\right),\\ a^{\langle i\rangle }=\Gamma_{o}*c^{\left(i\right)}. \end{array}$$

For ease of description, the LSTM in the example with this suction has only one branch. In actual applications, if there are multiple steps converging to one step, just add the corresponding *a* and *c* in the branched LSTM cell.

### 4.2. Output Sequence Structure in the Encoder-Decoder Model

The encoder can encode all input sequences *X*^(*i*)^ into a unified feature *c*. The decoder decodes it and outputs the results. We design the output as a fault type sequence. The value of a specific item represents the length of time before fault occurrence. This output model provides convenience for labelling training data.

The output is defined as a sequence of fault types. *y*^(*k*)^ is the output, and *k* is the type of fault. The value of *y*^(*k*)^ is the time length before the fault *k* occurs, but it is not a numerical value. We define it as a class set:

(10) $$\begin{array}{c} y^{\left(k\right)}=y_{1}^{\left(k\right)},y_{2}^{\left(k\right)},\ldots ,y_{i}^{\left(k\right)},\ldots ,y_{n}^{\left(k\right)}. \end{array}$$

Each element *y*_*i*_^(*k*)^ represents a time period before fault occurrence. Therefore, the output cell of the neural network is a SoftMax function. The advantage of this model is that when labelling the training data, it can roughly label the length of time before the fault cures. However, its drawback is that the length *n* of *y*^(*k*)^ and the time period represented by each *y*_*i*_^(*k*)^ depends on prior knowledge. Obviously, the model is a unidirectional propagation neural network.

### 4.3. AM for Identifying Factors

In the production process, the amount of data is very large. In other words, the input of the LSTM model is high-dimensional data. However, when faults occur, the data that it can affect may be only one or several dimensions. Therefore, most of the other data is redundant and ineffective. However, we do not know which data is redundant and which data is crucial. In this paper, we use an attention mechanism to identify the crucial data. There are at least two benefits. Firstly, LSTM is not good at handling a long sequence, and the AM algorithm can help LSTM deal with long sequence inputs to improve prediction accuracy. Secondly, the weight vectors in AM, originally used to identify crucial data, can be used to identify fault factors. It is helpful for the industry to improve the production process to prevent faults.

The attention mechanism has been widely used in the processing of various types of sequence data now. We firstly use AM in fault detection to handle the problem of overly long input sequences. Meanwhile, AM weight vectors can reflect the specific factors that cause the faults.

**Figure 7:**
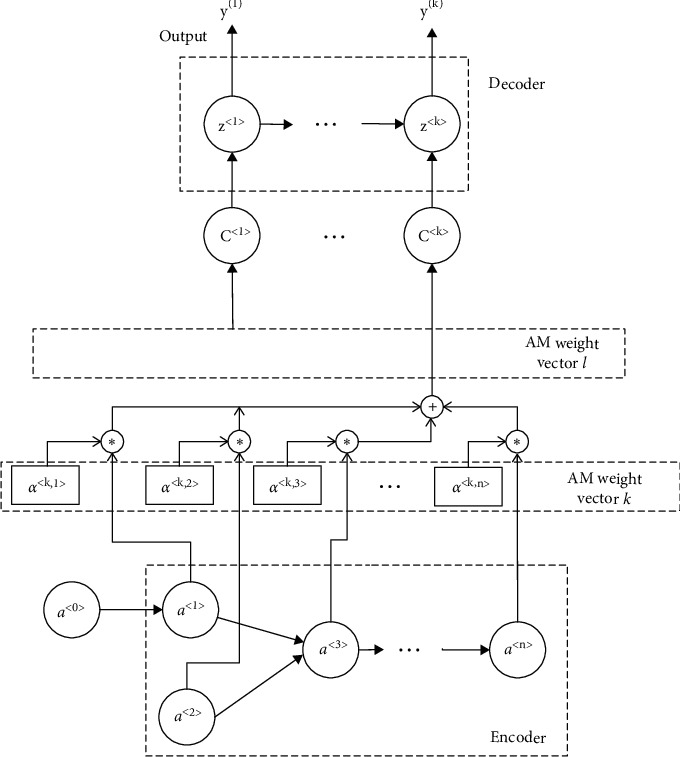
Branched LSTM-based AM structure in the encoder-decoder model.

The AM based on the encoder-decoder model is realized by adding an attention weight vector for each output. The outputs of every cell in the LSTM will combine the weight vector with the output features for the decoder. This is the same for the branched LSTM proposed in this paper. In other words, the encoder provides a feature vector for every output in each decoder instead of one single feature *c*. The structure is shown in Figure 7.

The AM in encoder provides a series of attention weight vectors, indicating the feature matrix. It can be described as

(11) $$\begin{array}{c} c^{\langle t\rangle }=\sum_{i=1}^{n}\alpha^{\langle t,i\rangle }a^{\langle i\rangle }, \end{array}$$

where *c*^〈*t*〉^ is the feature matrix for output *y*^〈*t*〉^ and *α*^{〈*t*, *i*〉}^ is a weight for sensor *i* in the attention weight vector *t*. *a*^〈*i*〉^ is the output of the cell *i*. *n* is the number of sensors.

Attention weights in one single vector need to meet constraints as follows:

(12) $$\begin{array}{c} \sum_{i=1}^{n}\alpha^{\langle t,i\rangle }=1. \end{array}$$

Attention weight *α*^〈*t*, *i*〉^ indicates the value of attention from output *y*^〈*t*〉^ paid to each activation value *a*^〈*i*〉^. *α*^〈*t*, *i*〉^ can be described as equation (13), which satisfies the constraint of equation (12):

(13) $$\begin{array}{c} \alpha^{\langle t,i\rangle }=\frac{\text{exp}\left(e^{\langle t,i\rangle }\right)}{\sum_{i=1}^{n}\text{exp}\left(e^{\langle t,i\rangle }\right)}, \end{array}$$

where *e*^〈*t*, *i*〉^ is calculated through the previous layer of LSTM neural networks.

After completing the design of neural networks, the details of a backpropagation algorithm and network training process can be found in [27, 37]. Attention weight vectors in trained networks can be used to identify the specific factors causing the specific faults.

## 5. Experiment and Evaluation

In this section, we apply the TEP to simulate the process model in MATLAB. Based on data from this model, some other fault detection and diagnosis algorithms are compared with the proposed approach.

### 5.1. Tennessee Eastman Process Model

**Figure 8:**
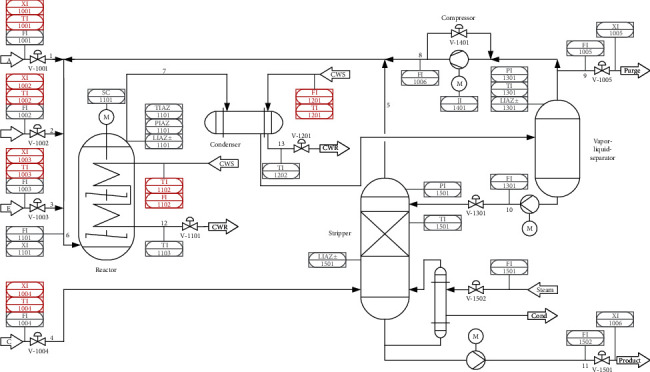
Flow sheet of the Tennessee Eastman benchmark process.

TEP is a well-known process simulation in the Chemical industry and is a benchmark of fault detection and diagnosis [3]. The latest revision of TEP was proposed in 2015, and there are more variables and types of faults exploded. The details and source code can be found in [4]. The piping and instrumentation diagram (P&ID) of the revised TEP simulator is shown in Figure 8.

The simulation model uses the input data from the definition of Downs and Vogel, including parameters and signals. The gaseous reactants A, C, D, and E and the inert B are fed to the reactor where the liquid products G and H are formed. The reactions in the reactor are

(14) $$\begin{array}{c} A\left(g\right)+C\left(g\right)+D\left(g\right)⟶G\left(\text{liq}\right),\\ A\left(g\right)+C\left(g\right)+E\left(g\right)⟶H\left(\text{liq}\right),\\ A\left(g\right)+E\left(g\right)⟶F\left(\text{liq}\right),\\ 3\;D\left(g\right)⟶2F\left(\text{liq}\right), \end{array}$$

where *g* and *lig* indicate raw material status.

In the simulator, there are 12 manipulated variables (MVs) considered as control signals. There are 41 measured variables, which can be seen as the sensing data in this proposed approach. In other words, they are the inputs of the encoder-decoder model. The first 22 were measured continuously and sampled every 3 min, XMEAS(l) through XMEAS(22), and they are listed in Table 1. The rest are composition measurements.

There were 21 different types of faults during production, named “Fault1, Fault2, … , Fault21.” We selected the first 20 faults. Their settings are found in [38]. We delayed the labelled time stamp by dozens of minutes for three faults. Some faults did not break production until after a period of time. The process data are 7670 hours in a fault state and 4000 hours in a normal state. The samples were randomly selected from process data. The total number of samples is 30,000. According to the encoder-decoder model, we randomly selected 80% of both fault and normal samples for the training dataset, and the remaining were used as the testing datasets. Descriptions of fault status are shown in Table 2.

### 5.2. Setup for the Encoder-Decoder Model

The input data came from 41 measured variables and 12 manipulated variables in the Tennessee Eastman Process (TEP) simulation, which entails 53-dimensional time series data. Therefore, the length of the input sequence for the encoder needed to be fixed at 53. Similarly, the length of outputs needed to be equal to the type number of faults, and situations with no fault detected indicated a normal status. In this simulation, it could be fixed to 21. The composition measurements from 41 measured variables were taken from Streams 6, 9, and 11. The sampling interval and time delay for Streams 6 and 9 were both equal to 6 minutes, and those for Stream 11 were equal to 15 minutes. All the process measurements included Gaussian noise. Based on the analysis of [39], we constructed the LSTM-based encoder-decoder model with one hidden layer.

The length of input data for one sensor, or single element in the input sequence, depends on the sampling time. It was empirically estimated. Its length needed to be greater than the duration before the faults broke production. In this simulation, we labelled several faults with time delays, which is illustrated in Table 2. Moreover, according to [38], within an hour, the time length is longer, and the accuracy of the deep learning algorithm classification is higher. We then set the max length of sampling time to 1 hour and tested the performance with less than 1 hour. According to the frequency of the sensor sampling frequency, the length of input data for one continuously measured variable was 20, and the discrete others were 10 or 4. These setups in the TEP model are described in Section 5.1. To facilitate matrix operations in deep learning, when the number of discrete samples was 4, only the first 3 data were taken. The output *y*^〈*i*〉^ of the decoder is the time length before fault *i* breaks production. The output layer was a SoftMax function, so *y*^〈*i*〉^ was not a continuous variable. When the value of the output sequence is maximum, the status is normal.

### 5.3. Evaluation

**Figure 9:**
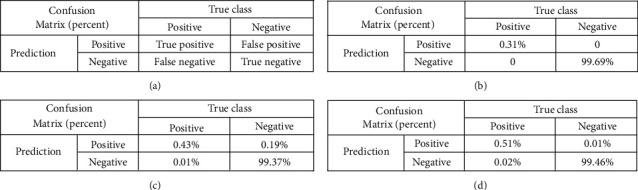
Confusion matrices. (a) Confusion matrix. (b) Confusion matrix of Fault1. (c) Confusion matrix of Fault9. (d) Confusion matrix of Fault17.

Each element in the output sequence is taken from a multiclassifier, and we used a multiclass evaluation indicator: macroaverage F1 score [40]. There were four possible results for fault *i* detection, and the detection result was a different time length *j* before the production break: (1) the result is positive, and the true value is positive too. The symbol used is TP_*j*_^(*i*)^ (True Positive for fault *i*, detection result *j*) representing the number of such results. (2) The result is positive, but the true value is negative. The symbol used is FP_*j*_^(*i*)^ (False Positive). (3) The result is negative, and the result is negative. The symbol used is FN_*j*_^(*i*)^ (False Negative). (4) The result is negative, but the true value is positive. The symbol used is TN_*j*_^(*i*)^ (True Negative). They are shown in Figure 9. Based on the definition above, we counted the TP_*j*_^(*i*)^, FP_*j*_^(*i*)^, and FN_*j*_^(*i*)^ for each type of fault. Afterwards, we calculated the precision and recall in equation (15). We also provide three confusion matrices of the typical Fault1, Fault9, and Fault17.

(15) $$\begin{array}{c} {\text{precision}}_{j}=\frac{{\text{TP}}_{j}^{\left(i\right)}}{{\text{TP}}_{j}^{\left(i\right)}+{\text{FP}}_{j}^{\left(i\right)}},\\ {\text{recall}}_{j}=\frac{{\text{TP}}_{j}^{\left(i\right)}}{{\text{TP}}_{j}^{\left(i\right)}+{\text{FN}}_{j}^{\left(i\right)}}. \end{array}$$

The F1-score, for every output from each fault type, can be described as

(16) $$\begin{array}{c} f1_{j}=\frac{2*{\text{precision}}_{i}*{\text{recall}}_{j}}{{\text{precision}}_{i}+{\text{recall}}_{j}}. \end{array}$$

The average of F1-score is

(17) $$\begin{array}{c} {\text{score}}^{\left(i\right)}={\left(\frac{1}{k}\sum_{j=1}^{k}f1_{j}\right)}^{2}, \end{array}$$

where *k* is the number of output classes in every element of the output sequence.

Table 3 shows the F1-score for each type of fault. There are low scores for identifying Fault15 and Fault16. The main reason is that the correlation between faults and sensing data is very low. Therefore, we considered them as exceptions and ignored their results. In fact, the F1-score should exceed 0.8 for the classifier to be considered acceptable. However, most data shown in Table 3 cannot satisfy it, since the correlation between the data and the faults is not all linearly related to the time before production break. The ultimate goal of fault detection is a biclassifier that detects whether a fault occurs. Thus, as the description above, we chose a threshold, which is used to convert the multiclassifier of a time length into a two-classifier; then, the model performs better. We display the performance of the approach proposed in this paper in Table 4, and it has been also compared with other approaches, including a basic Principle Component Analysis (PCA) method, a typical LSTM-based encoder-decoder structure, an optimized LSTM [41], and a Supported Vector Machine with a linear kernel and autoencoder method. We used the F1-score to evaluate them.

**Figure 10:**
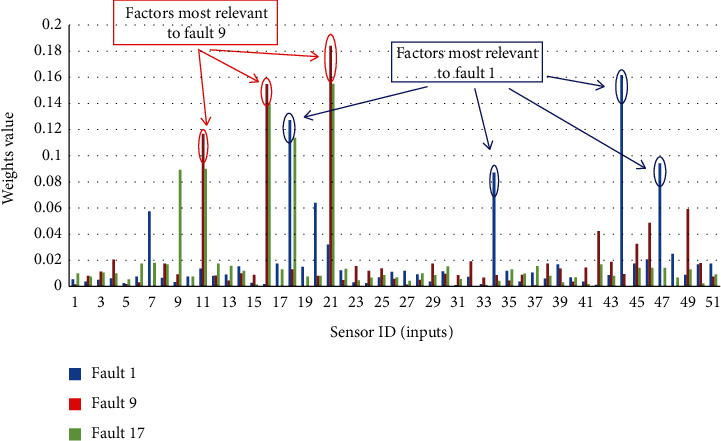
Identifying the factors causing fault by a weight vector.

As shown in the experimental results in Table 4, the traditional LSTM structure has a poor performance. The main reason is that the length of the input sequence is too long. A traditional LSTM structure lacks global information, and the update gate and forget gate in LSTM cells produce gradient disappearance during the propagation process. Only the autoencoder model performs better. AM can not only improve the accuracy of fault detection, shown in Table 4, but also identify the specific factors that cause the faults. In an encoder-decoder model with AM, each output *t* (meaning the fault *t*) is deduced by a specific feature matrix *c*^〈*t*〉^. *c*^〈*t*〉^ is calculated by all inputs and a weight vector *a*^〈*t*, *i*〉^. This structure is illustrated in Figure 7. The weight vector, that is, the attention weight, indicates the correlation of each input factor with fault *t*. In the experiments above, illustrated in Figure 10, we show weight vectors for some faults. The *x*-axis represents factors (i.e., sensors), and the *y*-axis represents weight values. Accordingly, we can identify the specific factors that cause the faults—factors with a high correlation will have high weights. For example, as shown in Figure 10, the specific factors that cause Fault9 are sensors with ID 21, 17, and 11.

## 6. Conclusions

The main goal of this paper is accurate fault prediction and cause identification in the industrial production process. We propose a new spatial input sequence, which is different from a traditional time sequence or time series data. This sequence can solve the problem of input dimension changes in a traditional time series; moreover, each element in the input sequence comes from a different production position, which will provide the possibility of identifying their correlation with faults. According to the spatial sequence, we propose branched LSTM to adapt to the branch structure in the production process. These structures are then used in an encoder-decoder model, and an AM algorithm is used to solve the problem of long sequence inputs. Finally, the weight vectors in AM can be used to indicate the correlation between input data and faults.

Experimental results show that the approach has the capability of identifying critical factors. It also has improved prediction accuracy. The main drawback of this approach is that an AM is complicated. The algorithm will occupy a large number of computing resources and has a poor real-time performance. Therefore, future work will focus on optimizing the model structure, making it more suitable for fault detection in industrial big data. Another drawback is that the output model requires prior data.

## Figures and Tables

**Table 1:** Process measurements.

Variables	Description	Units
XMEAS(l)	A feed (stream 1)	kscmh
XMEAS(2)	D feed (stream 2)	kg/hr
XMEAS(3)	E feed (stream 3)	kg/hr
XMEAS(4)	Total feed (stream 4)	Kscmh
XMEAS(5)	Recycle flow (stream 8)	Kscmh
XMEAS(6)	Reactor feed rate (stream 6)	Kscmh
XMEAS(7)	Reactor pressure	kPa gauge
XMEAS(8)	A feed (stream 1)	%
XMEAS(9)	Reactor temperature	Deg C
XMEAS(l0)	Purge rate (stream 9)	Kscmh
XMEAS(l1)	Product Sep temp	Deg C
XMEAS(l2)	Product Sep level	%
XMEAS(l3)	Prod Sep pressure	kPa gauge
XMEAS(l4)	Prod Sep underflow (stream 10)	m^3^/hr
XMEAS(l5)	Stripper level	%
XMEAS(l6)	Stripper pressure	kPa gauge
XMEAS(l7)	Stripper underflow (stream 11)	m^3^/hr
XMEAS(l8)	Stripper temperature	Deg C
XMEAS(l9)	Stripper steam flow	kg/hr
XMEAS(20)	Compressor work	kW
XMEAS(2l)	Reactor cooling water outlet Temp	Deg C
XMEAS(22)	Separator cooling water outlet temp	Deg C

**Table 2:** Fault description.

Fault index	Type	Time delay	Number of samples	Description
Fault1	Step	None	600	A/C feed ratio, B composition constant
Fault2	Step	None	600	B composition, A/C ratio constant
Fault3	Step	None	600	D feed temperature
Fault4	Step	40 minutes	600	Reactor cooling water inlet temperature
Fault5	Step	40 minutes	600	Condenser cooling water inlet temperature
Fault6	Step	40 minutes	600	A feed loss
Fault7	Step	40 minutes	700	C header pressure loss-reduced availability
Fault8	Random	40 minutes	600	A, B, C feed composition
Fault9	Random	40 minutes	600	D feed temperature
Fault10	Random	None	600	C feed temperature
Fault11	Random	None	600	Reactor cooling water inlet temperature
Fault12	Random	50	700	Condenser cooling water inlet temperature
Fault13	Slow drift	None	600	Reaction kinetics
Fault14	Sticking	40 minutes	600	Reactor cooling water valve
Fault15	Sticking	None	600	Condenser cooling water valve
Fault16	Unknown	40 minutes	700	Unknown
Fault17	Unknown	40 minutes	600	Unknown
Fault18	Unknown	None	600	Unknown
Fault19	Unknown	None	600	Unknown
Fault20	Unknown	None	600	Unknown
Normal	—	—	17700	A/C feed ratio, B composition constant

**Table 3:** F1-score from the testing dataset using the approach proposed in this paper.

States	Type	Maximum of F1-score	Minimum of F1-score	Average
Fault1	Step	0.91	0.78	0.84
Fault2	Step	0.85	0.71	0.78
Fault3	Step	0.67	0.53	0.6
Fault4	Step	0.98	0.95	0.97
Fault5	Step	0.77	0.62	0.71
Fault6	Step	0.99	0.95	0.97
Fault7	Step	0.99	0.96	0.98
Fault8	Random	0.89	0.78	0.83
Fault9	Random	0.7	0.51	0.6
Fault10	Random	0.98	0.92	0.96
Fault11	Random	0.991	0.975	0.982
Fault12	Random	0.86	0.78	0.8
Fault13	Slow drift	0.97	0.86	0.89
Fault14	Sticking	0.98	0.89	0.92
Fault15	Sticking	0.21	0.18	0.19
Fault16	Unknown	0.23	0.15	0.17
Fault17	Unknown	0.99	0.94	0.97
Fault18	Unknown	0.88	0.81	0.85
Fault19	Unknown	0.98	0.95	0.97
Fault20	Unknown	0.85	0.76	0.81

**Table 4:** Comparison fault detection result by F1-score.

States	PCA	Typical LSTM	Optimized LSTM	SVM using a linear kernel	Auto encoder	The proposed approach in this paper
Fault1	1	0.09	0.68	0.87	0.98	1
Fault2	0.79	0.12	0.78	0.88	0.85	0.89
Fault3	0.34	0.03	0.45	0.79	0.91	0.94
Fault4	0.99	0.04	0.75	0.9	0.89	0.99
Fault5	0.56	0.2	0.89	0.9	0.93	0.94
Fault6	0.99	0.34	1	0.95	0.85	1
Fault7	1	0.19	0.89	0.92	0.80	1
Fault8	0.97	0.22	0.71	0.63	0.73	0.99
Fault9	0.78	0.01	0.67	0.76	0.76	0.81
Fault10	0.66	0.28	0.77	0.89	0.79	0.99
Fault11	0.71	0.12	0.83	0.9	0.77	0.88
Fault12	0.99	0.31	0.56	0.75	0.93	0.99
Fault13	0.87	0.22	0.89	0.82	0.91	0.89
Fault14	0.98	0.41	0.99	0.88	0.78	0.99
Fault15	0.26	0.01	0	0.21	0.01	0.22
Fault16	0.24	0.12	0	0.14	0.33	0.31
Fault17	0.99	0.24	0.2	0.79	0.89	0.97
Fault18	0.78	0.31	0.88	0.66	0.95	0.89
Fault19	0.88	0.17	0.91	0.86	0.64	0.97
Fault20	0.82	0.28	0.64	0.78	0.83	0.85

## Data Availability

The data generated by the TEP simulation platform are used to support the findings of this study, and the method of obtaining data is described in detail within the article.
